# The Effect of Defect Charge and Parasitic Surface Conductance on Aluminum Nitride RF Filter Circuit Loss

**DOI:** 10.3390/mi14030583

**Published:** 2023-02-28

**Authors:** Tian Xu, Yali Zou, Xuan Huang, Junmin Wu, Shihao Wu, Yuhao Liu, Xuankai Xu, Fengyu Liu

**Affiliations:** 1 School of Microelectronics, Shanghai University, Shanghai 200444, China; 2 Changzhou Bawnovation Microelectronics Co., Ltd., Changzhou 213166, China; 3 School of Information Science and Technology, ShanghaiTech University, Shanghai 201210, China

**Keywords:** AlN, trap rich, polysilicon, CPW, RF loss

## Abstract

When AlN thin films are deposited directly on the high-resistance silicon (HR-Si) substrate, a conductive layer will be formed on the HR-Si surface. This phenomenon is called the parasitic surface conduction (PSC) effect. The presence of the PSC effect will increase the power consumption of electronic components. Therefore, it is necessary to reduce the PSC effect. In prior technology, the polysilicon layer is usually used as the trap-rich layer to reduce the PSC effect. Experiments show that compared to AlN films deposited directly on HR-Si, the AlN substrates with polysilicon introduced on HR-Si have less radio frequency (RF) loss. To verify the effect of polysilicon on RF loss, polysilicon films of three different thicknesses and several different roughnesses were introduced. The results show that the thickness of the polysilicon will affect the RF loss, while the roughness has almost no effect on it. The polysilicon trap-rich layer can reduce the RF loss, which gradually becomes smaller as the polysilicon thickness increases.

## 1. Introduction

The film bulk acoustic resonator (FBAR) is a sandwich structure consisting of metal electrode/piezoelectric layer/metal electrode; frequency regulation can be realized through the inverse piezoelectric effect of piezoelectric film [1], and a cavity is formed between the silicon wafer and the lower electrode [2]. As an ultra-wide bandgap semiconductor material, AlN has excellent piezoelectric properties, high acoustic surface wave rate, and a large electromechanical coupling coefficient, so it is often selected as the piezoelectric material of FBAR [3]. With the development of semiconductor technology, the excellent radio frequency (RF) characteristic of high-resistance silicon (HR-Si) is gradually discovered. It is shown that AlN films deposited directly on HR-Si have large RF losses, which is due to the large lattice mismatch and thermal mismatch between AlN and HR-Si, as well as the introduction of unintentional doping during the growth process [4,5]. It results in a large number of point defects in AlN materials, including Al vacancies and N vacancies which will form impurity scattering centers in the material and reduce carrier mobility and device performance while reducing its service life [6,7,8]. Due to the presence of charged point defects in AlN, a conductive layer is formed on the HR-Si surface, resulting in a parasitic surface conductivity effect (PSC). The existence of a conductive layer on the surface of HR-Si will reduce the effective resistivity of it and increase the high-frequency loss of the transmission line, thus resulting in larger RF losses and a poor device performance [9]. Therefore, in order to reduce the number of carriers between the AlN and the HR-Si substrate layer, while suppressing the PSC effect generated by the HR-Si substrate layer, researchers have tried various methods to improve the RF performance of the substrate [10,11]. The best method found so far is to introduce a carrier trap-rich trap layer on HR-Si. Due to the large number of grain boundaries and defects in polysilicon, it can effectively trap free carriers caused by the PSC effect, it is also compatible with mature integrated circuit (IC) manufacturing processes and has very good thermal stability [12]. Therefore, the PSC effect on HR-Si substrate can be effectively suppressed by the polysilicon carrier trap layer, and can further improve the RF performance of the substrate [13]. This method is currently widely used to eliminate the PSC effect on HR-Si on insulator (HR-SOI) substrates. The buried oxygen layer of the HR-SOI substrate contains a fixed charge, therefore, is subject to the PSC effect, resulting in a limitation of its RF performance [14]. In this paper, we try to introduce polysilicon between HR-Si and AlN film and verify the effect of polysilicon on RF loss by changing the thickness of polysilicon. After comparing the two commonly used transmission line structures, we chose to grow the coplanar waveguide (CPW) line on HR-Si to test its RF loss.

## 2. Thin Film Preparation and Characterization

### 2.1. Thin Film Preparation

**Figure 1:**
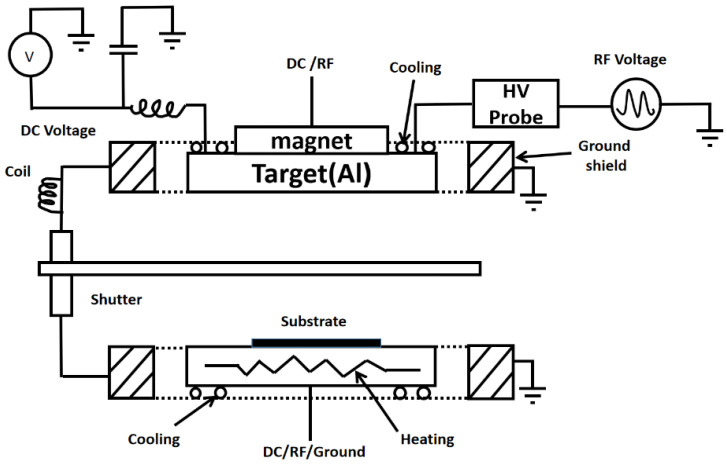
Principle of the AlN deposition.

In the experiment, six-inch HR-Si wafers were used, and the AlN thin films were prepared by the SPTS (KLA., Hayward, CA, USA) RF sputtering machine. Magnetron sputtering has the advantages of fast deposition rate, good compactness, good uniformity, and low roughness. The target material was an Al metal target with a purity of 99.9995%, the target-substrate separation distance was about 14 mm, and the gas pressure at work was about 3.6 × 10^−3^ Torr. After the system was pumped to a high vacuum, Argon gas was introduced, and a high voltage was applied between the cathode and anode. The phenomenon of glow discharge occurred between the two poles. The presence of an electric field attracted the positive ions generated by the discharge which then collided with the atoms on the surface of the Al target [15]. At the same time, the nitrogen required for the reaction was introduced into the sputtering chamber, and the Al atoms combined with nitrogen as they flew towards the substrate, eventually forming the AlN film covering the HR-Si surface [16]. Figure 1 shows the schematic diagram of magnetron sputtering.

In order to deposit a 1000 nm film, the deposition time for all wafers was set to 750 s. To ensure the uniformity of the film, the entire deposition process was divided into four steps, so the time of one step was 187.5 s, and each step rotated the wafer by 90° to prevent local unevenness. Since fast deposition is an advantage of magnetron sputtering, the time required was shorter than other methods. Sputtering power, gas flow, magnet rotation speed, etc., mainly affect the uniformity and stress of the film, while having little effect on the full width at half maximum (FWHM) and roughness. Therefore, we chose the best parameters of the machine to deposit AlN: the sputtering temperature was 300 °C, the sputtering power was 200 W, the density of sputtering power was 14 W/cm^2^, the total gas flow rate was 93 sccm, the nitrogen flow rate was 15.5 sccm, and the magnet rotation speed was 240 rpm.

Five six-inch HR-Si wafers were required for the experiment. Before the experiment, they were cleaned with a standard solution to remove possible particles on the surface. Wafer 1 was directly deposited with AlN, wafers 2 and 3 were first deposited in the furnace tube with 400 nm and 850 nm polysilicon, wafers 4 and 5 were deposited with 1150 nm polysilicon, wafers 2, 3, and 4 were polished with a chemical mechanical polishing (CMP) process after polysilicon was deposited, and their surface became smooth. The thickness of the polysilicon was reduced by 100 nm after the three wafers were polished by the same process. Table 1 shows the designed experimental scheme of the five wafers.

Compared with other chemical vapor deposition (CVD) methods, we chose to deposit polysilicon by the LPCVD furnace tube (Tempress. Epe, Vaassen, Netherlands) because the uniformity of LPCVD is better, the temperature control method is simpler, and the wafer is heated more evenly [17]. In this experiment, a horizontal furnace tube was used to prepare polysilicon; the wafers were put into the furnace tube stage, and then they were sent to the reaction chamber through the conveying system. The reaction gas was high-purity silane, and the carrier was pure nitrogen. The gas pressure was 20 mTorr, the silane flow rate was 200 sccm, and the temperature was 610 °C, while the time required for the experiment varied with the thickness of the polysilicon. Before the deposition started, a large amount of nitrogen gas was introduced; it can purge other residual gases in the furnace and can ensure that there is no interference from other gases during deposition. After that, the furnace was pumped to ensure that the furnace was always in a low-pressure environment, and to ensure the purity of the gas in the furnace. At the same time, nitrogen was constantly injected into the furnace when the wafers were retracted or the temperature was raised or lowered. This was to ensure a clean environment inside the furnace and prevent the wafers from being contaminated and oxidized by other gas during the growth process.

Due to the different thicknesses needed, several wafers cannot be deposited simultaneously with a furnace tube for polysilicon, and the polysilicon thickness can be changed by changing the deposition time. Since both polysilicon and monocrystalline silicon are composed of Si, the thickness of polysilicon deposited directly on the silicon wafer cannot be measured by an ellipsometer; thus, a 200 nm SiO_2_ wafer was placed at the same time before the polysilicon was deposited for a measurement of the thickness.

### 2.2. Thin Film Characterization

The thickness of polysilicon was measured by an ellipsometer and Scanning Electron Microscope-Focused Ion Beam (SEM-FIB) (Thermo Fisher Scientific, Waltham, MA, USA). The FWHM of AlN was analyzed by X-ray diffraction (XRD), and the roughnesses of polysilicon and AlN were compared by Atomic force microscopy (AFM) [18,19].

#### 2.2.1. Thin Film Thickness Characterization

**Figure 2:**
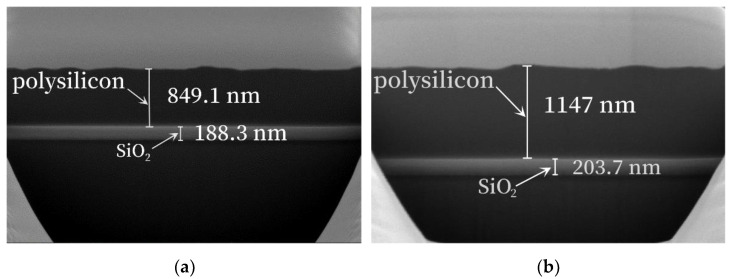
SEM-FIB thickness of polysilicon: (**a**) the actual thickness of polysilicon was estimated to be 850 nm; (**b**) the actual thickness of the polysilicon was estimated to be 1150 nm.

When measuring the thickness of polysilicon with an ellipsometer, it was found that the actual thickness of polysilicon, which was expected to be 400 nm, was 395 nm. The ellipsometer used the reflection of polarized light on the upper and lower surfaces of the film and obtained the relation between the optical parameters and the polarization state through the Fresnel formula to determine the refractive index and thickness of the optical film. This can be used for the nondestructive measurement of transparent films [20]. Therefore, as the thickness of the polysilicon increased, the film was gradually opaque, and polarized light could not be reflected by the film, thus the mean square error (MSE) was larger when measuring polysilicon with the expected thickness of 850 nm and 1150 nm. Thus, SEM-FIB was used to observe the thickness. The imaging principle of SEM was to use an electron beam as the illumination source, and the very finely focused electron beam was irradiated on the sample in a raster scanning manner. Through the interaction between electrons and specimens, secondary electrons, backscattered electrons, etc. were generated and then collected and processed to obtain a microscopic image of the magnified shape [21]. Figure 2a was the expected 850 nm polysilicon, and the actual thickness was 849 nm; Figure 2b was the expected 1150 nm polysilicon, and the actual thickness was 1147 nm. Therefore, the polysilicon thicknesses of wafer 2, wafer 3, wafer 4, and wafer 5 were 295 nm, 749 nm, 1047 nm, and 1147 nm, respectively.

#### 2.2.2. Roughness Characterization of Polysilicon and AlN

**Figure 3:**
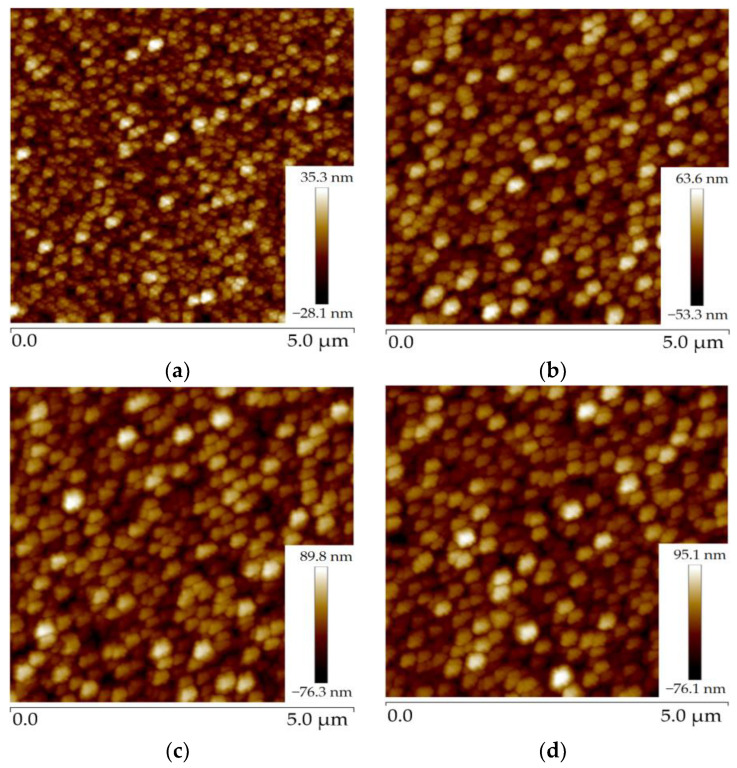
Figure of roughness of four pieces of polysilicon: (**a**) polysilicon roughness of wafer 2; (**b**) polysilicon roughness of wafer 3; (**c**) polysilicon roughness of wafer 4; (**d**) polysilicon roughness of wafer 5.

AFM uses a micro-cantilever to sense the interaction between the tip of the needle and the sample, thus achieving the purpose of detection with atomic resolution [22]. The micro-cantilever corresponds to the change in position of each point of the scan, so that information on the surface morphology of the sample can be obtained [23]. The polysilicon deposited in the furnace tube had a large roughness. The roughnesses of the second to fifth wafers observed by AFM were 6.91 nm, 13.3 nm, 19 nm, and 18.9 nm, respectively. Figure 3a–d show the AFM images of wafers 2, 3, 4, and 5, respectively.

**Figure 4:**
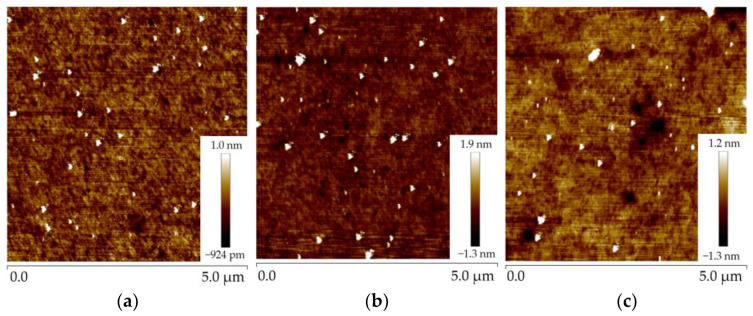
Figure of the roughness of three pieces of polysilicon: (**a**) the roughness of polysilicon after CMP of wafer 2; (**b**) the roughness of polysilicon after CMP of wafer 3; (**c**) the roughness of polysilicon after CMP of wafer 4.

As can be seen from the figure, the thicker the polysilicon, the higher the roughness. After the CMP process, the roughness of the polysilicon was significantly decreased, and the roughnesses of the first three pieces were reduced to 0.182 nm, 0.231 nm, and 0.226 nm. Figure 4 displays the AFM images of the three pieces of polysilicon. It can be seen that the CMP process can effectively reduce the roughness of the film.

**Figure 5:**
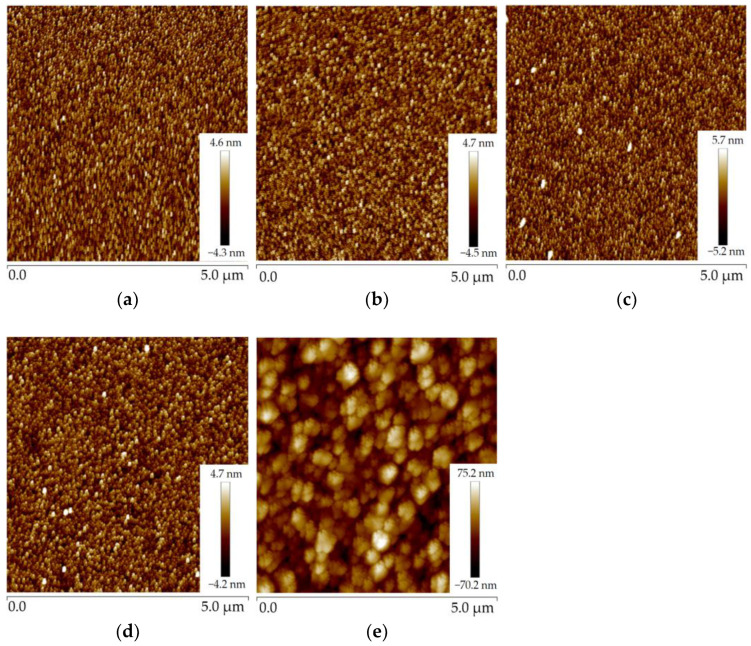
Figure of AlN wafer roughness: (**a**) AlN roughness image of wafer 1; (**b**) AlN roughness image of wafer 2; (**c**) AlN roughness image of wafer 3; (**d**) AlN roughness image of wafer 4; (**e**) AlN roughness image of wafer 5.

The 1000 nm AlN films were deposited simultaneously on five wafers. Figure 5 shows the AFM images of AlN on wafers 1 to 5; the roughnesses are 1.04 nm, 1.06 nm, 1.21 nm, 0.992 nm, and 17.2 nm, respectively. It can be found that the roughness of AlN deposited on polysilicon without CPM is the largest, and the roughness of AlN deposited on polysilicon with CMP is not much different from that of AlN without polysilicon.

### 2.3. Measurement of FWHM of AlN

**Figure 6:**
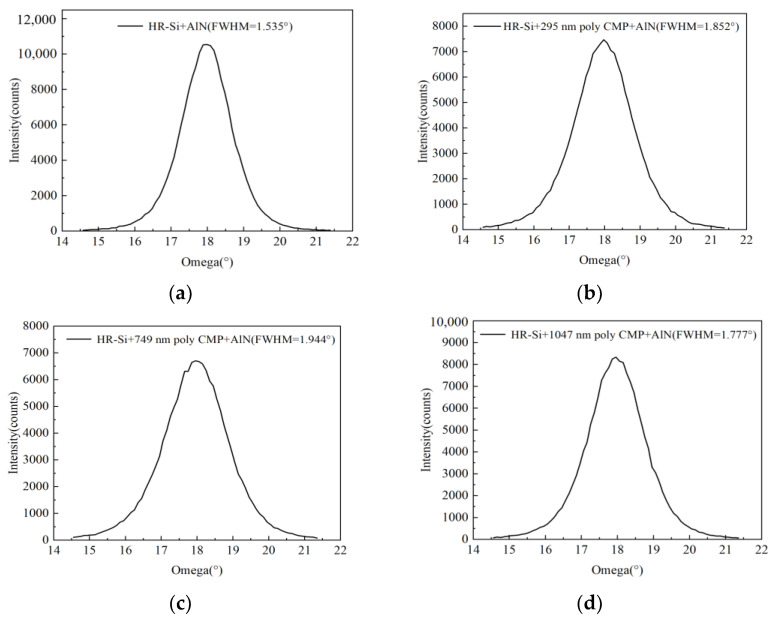
FWHM images of AlN: (**a**) FWHM of wafer 1; (**b**) FWHM of wafer 2; (**c**) FWHM of wafer 3; (**d**) FWHM of wafer 4.

Firstly, the ω-2θ of AlN was scanned; the scanning result can be located on the diffraction peak of the (002) crystalline plane when 2θ = 36.04°. The rocking curve of the AlN crystal plane was tested by XRD (Malvern Panalytical. Almelo, Netherlands). The FWHM of the rocking curve can reflect the c-axis consistency of the AlN piezoelectric film [24]. The smaller the FWHM, the better the c-axis consistency and crystalline phase of AlN piezoelectric films. The FWHM of AlN measured by XRD were 1.535°, 1.852°, 1.944°, and 1.777°. Because the roughness of the polysilicon without CMP was too large, the AlN film deposited on it had no crystalline phase, so the FWHM could not be measured. Figure 6 shows the FWHM of AlN.

## 3. RF Characterization

### RF Loss Analysis

The most common transmission line structures on HR-Si substrates are coplanar waveguide (CPW) and microstrip (MS). The signal and ground of the microstrip line are located on the surface and backside of the silicon substrate, which is small in size, light in weight, wide in a frequency band, high in reliability, and low in manufacturing cost. However, it has a slightly higher loss and a smaller power capacity. The ground and signal lines of the coplanar waveguide structure are in the same plane, and its electric field is parallel to the surface of the substrate, mainly concentrated on the silicon substrate surface [25]. According to the information available, the penetration depth of HR-Si and standard silicon is 6.5 times and 3 times the width of the CPW groove, respectively. Moreover, there will be an ultra-high conductive layer on the surface of HR-Si that is ten to one hundred times higher than that of standard silicon. This property of it causes the electric field to concentrate almost entirely in this conductive layer, resulting in greatly increased losses; this characteristic of CPW is just suitable for studying the PSC effect between HR-Si and AlN. At the same time, because CPW is a passive linear device and does not generate harmonics, it is an excellent choice for evaluating the harmonic level of the substrate [26]. Since the CPW is a planar structure and the manufacturing process is very simple, we chose the CPW structure to explore the RF loss generated between HR-Si and AlN.

**Figure 7:**
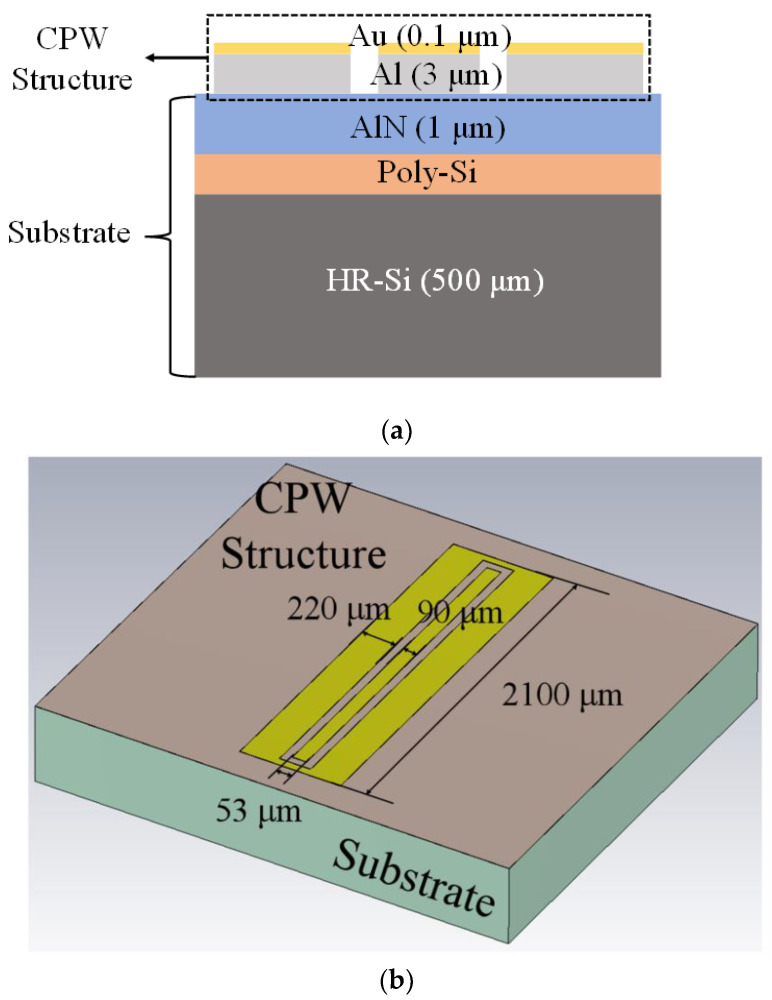
(**a**) Cross-section of the CPW structure; (**b**) 3D diagram of the CPW structure.

CPW wire is grown on the surface of the wafer, composed of 3 μm metal Al and 0.1 μm Au. The purpose of adding Au is to prevent Al from being oxidized into alumina by air. The resistance of alumina greatly affected the test results. Figure 7a shows the structure diagram of the CPW line. The thinnest line in the middle is the signal line, and the symmetric line with a slightly thicker width on both sides is the ground line. Figure 7b shows the dimensions of the CPW. The width of the signal cable is 90 μm, the width of the ground cable is 220 μm, and the length of the CPW is 2100 μm.

**Figure 8:**
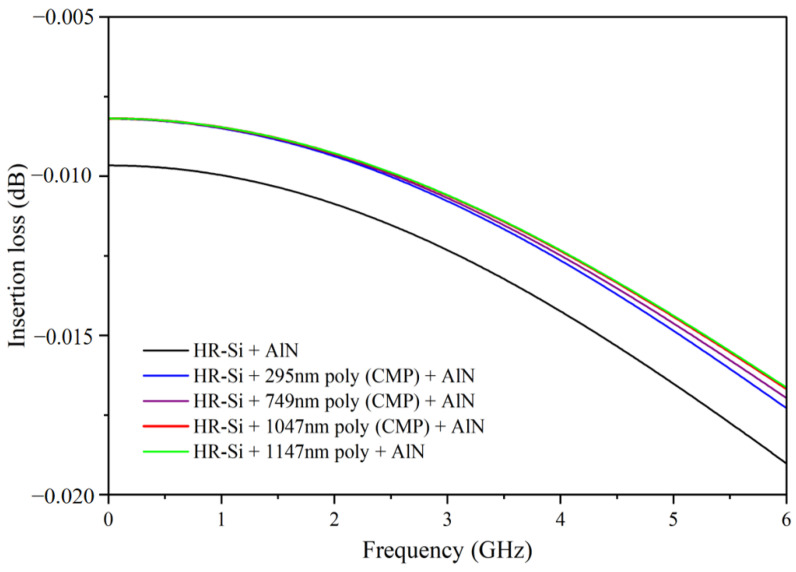
Simulation diagram of RF loss of high resistance silicon under different conditions.

Simulations were conducted using CST to evaluate the performance of coplanar waveguides (CPW) on various substrates. The simulated CPW had open boundaries in all directions and a load of 50 Ω at both port 1 and port 2. To mimic the proximity-induced surface conductivity (PSC) effect, a lossy layer was added between the AlN and HR-Si layers when there was no polysilicon present. This lossy layer had a dielectric constant of 4.5 and a conductivity of 1 S/m. As can be seen in Figure 8, the simulation results indicate that the absence of polysilicon deposition results in the highest RF loss due to the PSC effect, while the presence of polysilicon reduces the RF loss as its thickness increases.

**Figure 9:**
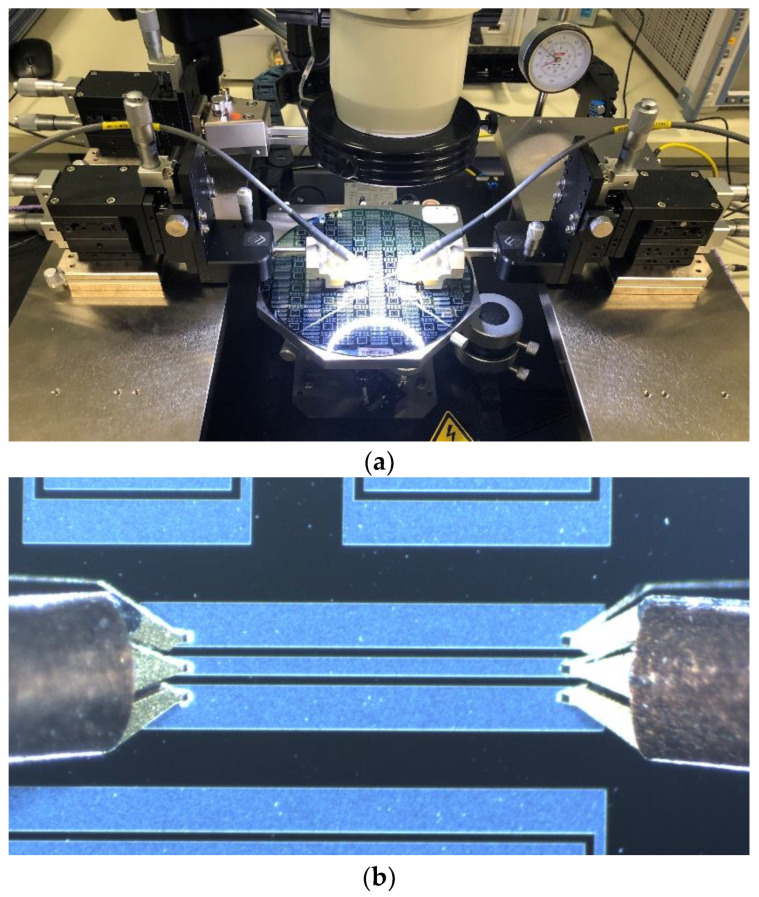
Experiment configuration: (**a**) wafer probe station; (**b**) the tested CPW.

The experiment was conducted using a vector network analyzer (VNA) to test 3001 points in the frequency range of 0.01 GHz to 6.01 GHz on a CPW using GSG probes. The experiment configuration is illustrated in Figure 9.

**Figure 10:**
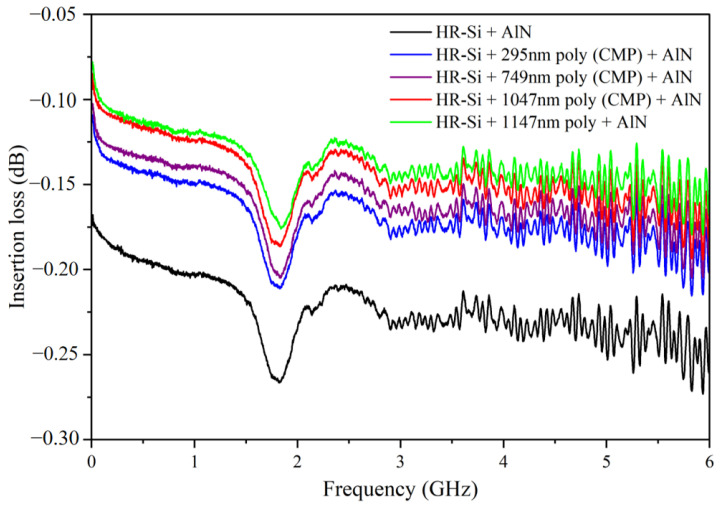
RF loss of high resistance silicon under different conditions.

The results shown in Figure 10 match the simulation predictions. A resonance at 1.8 GHz was observed, which was determined to be caused by the bulk acoustic wave (BAW) resonance of AlN due to its piezoelectric effect. This resonance was not present in the simulations because the CST simulations only considered the permittivity and conductivity of the materials. Based on the experimental results, it can be confirmed that the PSC effect between AlN and HR-Si will cause extra RF loss; introducing polysilicon between AlN and HR-Si can help improve the RF performance. The loss of undeposited polysilicon, deposited 295 nm, 749 nm, 1047 nm, and 1147 nm polysilicon substrate are −0.23 dB, −0.18 dB, −0.16 dB, −0.15 dB, and −0.14 dB, respectively. By comparing the RF loss of 295 nm, 749 nm, and 1047 nm polysilicon, it can be found that the RF loss decreases gradually with the increase in the thickness of the polysilicon. In addition, it can be found that compared with 1147 nm polysilicon, the roughness of 1047 nm polysilicon is much smaller, but the RF loss of 1047 nm polysilicon is still larger than it, thus indicating that roughness has little effect on RF loss, and the main factor affecting RF loss is still the thickness of polysilicon.

## 4. Conclusions

In this paper, a low-loss HR-Si substrate was obtained by depositing a layer of polysilicon between HR-Si and AlN. However, according to the experimental results, the roughness of polysilicon increases with the thickness. Moreover, the roughness of polysilicon affects the crystalline phase of AlN. Therefore, in order to obtain high-quality AIN films, the roughness of polysilicon must be reduced. For this purpose, the CMP process was introduced, and the roughness of polysilicon was reduced from about 10 nm to about 1 nm after the CMP process. The deposited AlN film also changed from a crystal-free phase to a half-peak width of about 1.8°. By comparing the experimental results of testing the RF loss, it was found that the thicker the polysilicon, the smaller the RF loss. When the thickness of the deposited polysilicon was increased from 295 nm to 1147 nm, the RF loss was reduced from −2.2 dB to −1.7 dB, which significantly improved the RF performance.

## Figures and Tables

**Table 1:** The five-wafer experiment scheme.

	Polysilicon Thickness	Whether CMP	AlN Thickness
Wafer 1	/	No	1000 nm
Wafer 2	400 nm	Yes	1000 nm
Wafer 3	850 nm	Yes	1000 nm
Wafer 4	1150 nm	Yes	1000 nm
Wafer 5	1150 nm	No	1000 nm

## Data Availability

Not applicable.
